# A Subset of Protective γ_9_δ_2_ T Cells Is Activated by Novel Mycobacterial Glycolipid Components

**DOI:** 10.1128/IAI.01322-15

**Published:** 2016-08-19

**Authors:** Mei Xia, Danny C. Hesser, Prithwiraj De, Isaac G. Sakala, Charles T. Spencer, Jay S. Kirkwood, Getahun Abate, Delphi Chatterjee, Karen M. Dobos, Daniel F. Hoft

**Affiliations:** a Division of Infectious Diseases, Allergy & Immunology, Edward A. Doisy Research Center, Saint Louis University School of Medicine, St. Louis, Missouri, USA; b Department of Microbiology, Immunology, and Pathology, Colorado State University, Fort Collins, Colorado, USA; c Proteomics and Metabolomics Facility, Colorado State University, Fort Collins, Colorado, USA; Weill Cornell Medical College

## Abstract

γ_9_δ_2_ T cells provide a natural bridge between innate and adaptive immunity, rapidly and potently respond to pathogen infection in mucosal tissues, and are prominently induced by both tuberculosis (TB) infection and bacillus Calmette Guérin (BCG) vaccination. Mycobacterium-expanded γ_9_δ_2_ T cells represent only a subset of the phosphoantigen {isopentenyl pyrophosphate [IPP] and (*E*)-4-hydroxy-3-methyl-but-2-enylpyrophosphate [HMBPP]}-responsive γ_9_δ_2_ T cells, expressing an oligoclonal set of T cell receptor (TCR) sequences which more efficiently recognize and inhibit intracellular Mycobacterium tuberculosis infection. Based on this premise, we have been searching for M. tuberculosis antigens specifically capable of inducing a unique subset of mycobacterium-protective γ_9_δ_2_ T cells. Our screening strategy includes the identification of M. tuberculosis fractions that expand γ_9_δ_2_ T cells with biological functions capable of inhibiting intracellular mycobacterial replication. Chemical treatments of M. tuberculosis whole-cell lysates (MtbWL) ruled out protein, nucleic acid, and nonpolar lipids as the M. tuberculosis antigens inducing protective γ_9_δ_2_ T cells. Mild acid hydrolysis, which transforms complex carbohydrate to monomeric residues, abrogated the specific activity of M. tuberculosis whole-cell lysates, suggesting that a polysaccharide was required for biological activity. Extraction of MtbWL with chloroform-methanol-water (10:10:3) resulted in a polar lipid fraction with highly enriched specific activity; this activity was further enriched by silica gel chromatography. A combination of mass spectrometry and nuclear magnetic resonance analysis of bioactive fractions indicated that 6-*O*-methylglucose-containing lipopolysaccharides (mGLP) are predominant components present in this active fraction. These results have important implications for the development of new immunotherapeutic approaches for prevention and treatment of TB.

## INTRODUCTION

Tuberculosis (TB), caused by Mycobacterium tuberculosis, remains a global health crisis and has become increasingly prevalent and deadly as a result of the emergence of both multidrug-resistant (MDR) TB and the HIV/AIDS pandemic (1). Given the evidence that TB patients develop immune dysfunction, immune intervention regulating counterproductive inflammation and/or enhancing anti-TB immune responses may be beneficial for clinical treatment of TB and MDR TB (2, 3). While immune therapy has been long considered attractive for adjunctive clinical treatment of TB (especially MDR TB); successful immunotherapeutic modalities have not been identified (4–6). In fact, little is known about human TB immunity except that human CD4^+^ T cells are generally believed to be important for TB resistance (2, 7–10) Accumulating evidence suggests that γδ T cells play a critical role in TB protective immune responses and may be key both as early responders to acute infection and for bridging innate and adaptive immunity (2, 11–18).

Unlike conventional major histocompatibility complex (MHC)-restricted CD4^+^ and CD8^+^ αβ T cells, human and nonhuman primate γ_9_δ_2_ T cells are stimulated to expand in a non-MHC-restricted way by small phosphorylated nonproteinaceous antigens (e.g., IPP, BrHPP, HMBPP, and TUBAg2 to -4; see below) (19–22). Natural phosphoantigens, including prenyl pyrophosphate synthetic precursors of lipid and steroid biosynthesis and phosphorylated nucleotides, are secreted in increased amounts as by-products from infecting pathogens and host cells altered by infection (23–25). The reactivity of γ_9_δ_2_ T cells with non-MHC-restricted, nonvariable M. tuberculosis ligands provides the potential advantage of universal subject responsiveness regardless of complex HLA expression patterns in human populations.

Specific to M. tuberculosis, earlier biochemical analysis identified 4 molecules from M. tuberculosis lysates, termed TUBag1 to -4, that stimulated the proliferation of a human γ_9_δ_2_ T cell clone (G115) (26). These TUBag compounds have been shown to be active in the nanomolar range (i.e., with bioactivities up to 30,000-fold higher than that of isopentenyl pyrophosphate [IPP]), thus suggesting that these molecules could account for most of the γ_9_δ_2_ T cell-stimulating activity recovered from mycobacteria. Shortly after isolation of TUBag1 to -4, the first γ_9_δ_2_ T cell antigen structurally identified was IPP, a metabolite all organisms use to synthesize isoprenoid compounds. The prenyl pyrophosphate family of phosphoantigens includes isomers, conjugates, or concatemers of IPP (27). Exchange of the pyrophosphate moiety for a single phosphate moiety significantly reduces the ability of these isoprenoids to stimulate the expansion of γ_9_δ_2_ T cells (23, 28). In contrast, alteration of the alkyl chain or conjugation to UTP had only minor influences on the potency of these phosphoantigens to expand γ_9_δ_2_ T cells, suggesting that IPP is the naturally occurring prenyl pyrophosphate capable of stimulating γ_9_δ_2_ T cell expansion. However, the concentration of IPP present in bacterial lysates is not sufficient to stimulate γ_9_δ_2_ T cell expansions (29). The most potent natural phosphoantigen described so far is a phosphorylated intermediate produced by Eubacteria and some eukaryotic organisms, called (*E*)-4-hydroxy-3-methyl-but-2-enylpyrophosphate (HMBPP, also known as HDMA-PP for hydroxy-dimethyl-allyl-pyrophosphate). This compound is 10,000 to 30,000-fold more potent for stimulation of γ_9_δ_2_ T cells than IPP (30, 31). In addition, synthetic bromohydrin pyrophosphate (BrHPP) is a strong activator of γ_9_δ_2_ T cells and has been tested for tumor immunotherapy and as a vaccine component (21, 32–34).

As demonstrated by us previously, live mycobacteria and purified prenyl pyrophosphates induce similar expansions of γ_9_δ_2_ T cells capable of gamma interferon (IFN-γ) production and cytolytic activity, while γ_9_δ_2_ T cells stimulated with IPP, BrHPP, or HMBPP fail to inhibit intracellular mycobacterial growth even after concomitant stimulation with Toll-like receptor (TLR) ligands (35). We found that γ_9_δ_2_ T cells capable of inhibiting intracellular M. tuberculosis growth represent only a subset of the phosphoantigen (IPP and HMBPP)-responsive γ_9_δ_2_ T cells. This protective subset expresses a more oligoclonal set of T cell receptor (TCR) sequences, capable of pathogen-specific recognition of M. tuberculosis-infected human macrophages. We hypothesized that the high potency of natural HMBPP phosphoantigen present in mycobacterial extracts could obscure detection of the protective γ_9_δ_2_ T cell subset. We also hypothesize that live mycobacteria express antigens (Ags), other than the previously identified phosphoantigens, which are required to induce the subset of γ_9_δ_2_ T cells protective against M. tuberculosis intracellular replication. These natural TB-specific γ_9_δ_2_ T cell Ags must be identified and purified for use as optimal immunotherapies or vaccines targeting the protective subset of γ_9_δ_2_ T cells.

In this study, we have established a novel strategy to fractionate and biochemically treat mycobacterial lysates to identify the molecule(s) responsible for the expansion of γ_9_δ_2_ T cells capable of inhibiting intracellular mycobacterial growth. We first ruled out protein, nucleic acid, and apolar lipids with basic separations and enzymatic digestions. Mild acid hydrolysis, which digests complex carbohydrate structures, had the largest effect on specific activity. Fractions derived from a 10:10:3 chloroform-methanol-water extraction of M. tuberculosis H37Rv cells primarily consisted of glycolipids and carbohydrates. Antigenic fractions were tested for the ability to clonally expand human γδ T cells with inhibitory activity for intracellular mycobacteria. The highest biological specific activity (SA) was found in the most polar fractions eluted off silica columns with 100% methanol. Further fractionation using size exclusion column chromatography (G-50 column) was utilized and demonstrated the highest activity in an early-eluting fraction. These complex, antigenically active fractions were analyzed via matrix-assisted laser desorption ionization–time of flight (MALDI-TOF) mass spectrometry (MS), thin-layer chromatography (TLC), ^1^H nuclear magnetic resonance (NMR), and gas chromatography-mass spectrometry (GC-MS). These analyses revealed that methylglucose lipopolysaccharides (mGLP) are the predominant components present in all of the most highly active fractions. Further identification, purification, and synthesis of the novel mycobacterial lipid components which induce TB protective γ_9_δ_2_ T cells may result in new immune intervention strategies for sensitive and drug-resistant TB.

## MATERIALS AND METHODS

### Isolation of PBMCs and monocytes.

Human peripheral blood mononuclear cells (PBMCs) were obtained from healthy tuberculin skin test-positive donors by leukapheresis. Monocytes were purified from PBMCs by plastic adherence based on the unique adhesion properties of monocytes/macrophages among PBMC populations (36–38).

### Media and reagents.

Mammalian cell culture experiments were completed and Mycobacterium bovis BCG stocks prepared as reported previously (35, 39). Whole-cell lysates of M. tuberculosis (MtbWL) (NR-14822) were obtained from BEI Resources. Soluble 4-hydroxy-but-2-enyl pyrophosphate (HMBPP; Echelon Bioscience, Salt Lake City, UT) was used for stimulation of γ_9_δ_2_ T cells as a control in inhibition assays. Interleukin 2 (IL-2; Hoffmann-LaRoche Inc., Basel, Switzerland) was used to expand γ_9_δ_2_ T cells. Anti-CD3 peridinin chlorophyll protein (PerCP; clone SK7), anti-αβ TCR fluorescein isothiocyanate (FITC; clone B3), anti-γδ TCR phycoerythrin (PE; clone 11F2), anti-CD4 PE-cy7 (clone RPA-T4), anti-CD8 V500 (clone RPA-T8), anti-Ki-67 (clone 35/Ki-67), anti-tumor necrosis factor alpha (anti-TNF-α; MAb11), anti-IFN-γ (B27), and anti-granzyme A (anti-GzmA; CB9) were all from BD Biosciences. Guava ViaCount Flex reagent (Millipore; 4700-0060) was used for accurate counting and discrimination of viable and nonviable cells from antigen-expanded cells. All chemicals for biochemical separations were obtained from Acros Organics (silica gel 60) and Sigma-Aldrich (chloroform and methanol). Alkaline phosphatase (AP) was obtained from Fisher Scientific (BP 80975).

### Purification and characterization of the novel lipid components from M. tuberculosis.

Total lipid was extracted from H37Rv gamma-irradiated, lyophilized cells with CHCl_3_-CH_3_OH-H_2_O (10:10:3 [vol/vol/vol]) for 2 h at room temperature, with thorough mixing. The supernatant was dried under nitrogen. Dry-cell extract (2 g; ∼1.5 g of carbohydrate content by α-naphthol) was used to coat silica gel (60 to 200 μm, 60 Å; 2 g) and loaded (dry) in a silica gel column (60 to 200 μm, 60 Å; 40 g). The column was eluted with CHCl_3_ (60 ml), 20% (vol/vol) MeOH in CHCl_3_ (60 ml), 40% (vol/vol) MeOH in CHCl_3_ (60 ml), 60% (vol/vol) MeOH in CHCl_3_ (60 ml), 80% (vol/vol) MeOH in CHCl_3_ (60 ml), and MeOH (60 ml). A quick α-naphthol test revealed that the 40 to 100% MeOH-CHCl_3_ fractions were carbohydrate rich. Carbohydrate contents were approximately 336 mg (40% MeOH), 420 mg (60% MeOH), 360 mg (80% MeOH), and 216 mg (100% MeOH). Aliquots (5 μl) from each of the fractions (40 to 100%) were taken, and an alditol acetate assay was performed using GC-MS.

### Treatment of mycobacterial lysates. (i) Reduction and alkylation.

Lysate was suspended at 1 mg/ml in 6 M guanidine hydrochloride and 0.6 M Tris-HCl, pH 8.6. Fresh 4 M dithiothreitol (DTT) was added to a final concentration of 4 mM, and the sample was incubated with stirring at 25°C for 3 h. Fresh 160 mM iodoacetamide (IAA) was added to a final concentration of 5 mM, and the sample was incubated at 37°C for 30 min. Samples were dialyzed into 10 mM ammonium bicarbonate to remove excess DTT and IAA.

### (ii) Delipidation.

Lysate was delipidated by two extractions with a 2:1 chloroform-methanol solution for 2 h at 25°C, followed by centrifugation at 27,000 × *g* for 1 h. The organic layer was removed. The remaining pellet was further extracted two additional times using a 10:10:3 solution of chloroform-methanol-water and centrifuged as before. The final insoluble pellet was used as the total delipidated mycobacterial fraction after being dried under nitrogen to remove residual solvent.

### (iii) DNase and RNase treatment.

Lysate was suspended in Tris-HCl buffer containing 5 mM magnesium chloride. DNase and RNase were added to a final concentration of 0.05 mg/ml each, and the samples were incubated at 4°C for 1 h. Enzymes were inactivated by multiple freeze-thaw cycles.

### (iv) Pronase digestion.

Lysate was suspended at 1 mg/ml in 0.2 M ammonium bicarbonate. Pronase stock solution, at 1 mg/ml in 0.2 M ammonium bicarbonate, was added to a final concentration of 0.1 mg/ml, giving an enzyme-to-protein ratio of 1:10. The samples were incubated overnight at 37°C; pronase was inactivated by multiple freeze-thaw cycles, and peptides were separated from undigested material by passing through a membrane with a 10-kDa cutoff.

### (v) Trypsin digestion.

Lysate was suspended at 1 mg/ml in 0.2 M ammonium bicarbonate. Trypsin stock solution, at 0.08 mg/ml in 0.2 M ammonium bicarbonate, was added to final concentration of 0.02 mg/ml, giving an enzyme-to-protein ratio of 1:50. The samples were incubated overnight at 37°C. Trypsin was inactivated by addition of trifluoroacetic acid (TFA; 10%) to 0.5%, and peptides were separated from undigested material by passing through a membrane with a 10-kDa cutoff, followed by drying on a SpeedVac to remove residual TFA.

### (vi) Mild acid hydrolysis.

Lysate was suspended at 10 mg/ml in 2 M trifluoroacetic acid, incubated at 120°C for 2 h, cooled, and dried on a SpeedVac. The dry material was suspended in water.

### (vii) Base hydrolysis.

Lysate was suspended at 10 mg/ml in 0.15 M Tris-HCl, pH 8, to which NaOH was added to 0.16 M. The sample was then incubated at 37°C for 2 h. TFA (10% solution) was added to a final concentration of 1% to stop the reaction. Samples were dried on a SpeedVac, and the dried material was suspended in water.

### Resolution of 100% MeOH fraction for mGLP enrichment.

A dry 100% MeOH fraction (12 mg of carbohydrate; 25-mg stoichiometric weight) was dissolved in water (0.5 ml), loaded onto the size exclusion column (G-50; 114 cm by 0.75 cm), and eluted with water. The flow rate was 0.55 ml/min. Fractions (120; each 2.5 ml per fraction/5.0 min) were collected. A quick α-naphthol assay was performed to identify the carbohydrate-enriched (25th to 43th; 62.5 to 107 ml) fractions. Every three consecutive fractions were then pooled, and the monosaccharide composition was determined using GC-MS. The analysis revealed that the 62.5- to 70-ml fraction had the most pure and enriched mGLP content (yield: 250 μg).

### MALDI-TOF MS.

Purified silica gel column fractions (1 μl) were mixed with 1 μl of 2,5-dihydroxybenzoic acid (DHB; 10 mg/ml in 50% acetonitrile–0.1% TFA). Mixtures were allowed to dry on the MALDI target plate. Samples were analyzed by an Ultraflex-TOF/TOF mass spectrometer (Bruker Daltonics) in negative-ion, reflective mode, using a 25-kV accelerating voltage, and FlexAnalysis software was used to generate the spectra.

### Monosaccharide composition.

Aliquots of G-50 column eluates were hydrolyzed with 2 M trifluoroacetic acid, converted to alditol acetates, and analyzed using GC-MS performed as described previously (40).

### ^1^H and ^31^P NMR analysis.

Both ^1^H and ^31^P NMR were recorded on a 400-MHz Innova400 (Varian). NMR analysis of the 100% CH_3_OH eluate was performed on 4.0 mg of material. The fraction was first dried in a 13- by 100-mm glass tube under a stream of N_2_, D_2_O exchanged, dried, and reconstituted in 600 μl of D_2_O prior to the NMR analysis. The ^1^H NMR was recorded in PRESAT mode with 128 scans, and the ^31^P NMR was recorded with 9,900 scans. The ^31^P NMR of commercial HMBPP (2 mg in 600 μl of D_2_O) was also recorded with 1,024 scans.

### AP assay.

H37Rv total lipid from the extraction with CHCl_3_-CH_3_OH-H_2_O (10:10:3) was dried and resuspended in CHCl_3_-CH_3_OH (2:1), and the mixture was centrifuged at 3,600 × *g* and 4°C for 10 min. The insoluble material was resuspended in H_2_O and filtered with an Amicon Ultra (3,000-molecular-weight cutoff [MWCO]) iteratively to accumulate both retentate and eluate substrates for alkaline phosphatase treatments. A 1.0-mg quantity of retentate or eluate was treated with 400 U of AP in Optizyme buffer at 37°C for 1 h, and reactions were stopped by incubation at 70°C for 5 min. Sham reactions included AP without a substrate and either retentate or eluate without AP. Dephosphorylated samples were exchanged into D_2_O for evaluation with ^31^P NMR.

### Thin-layer chromatography.

High-performance thin-layer chromatography sheets (EMD; F_254_; silica gel 60) were used to analyze silica column fractions. We applied 20-μg fractions per lane and developed the plates with chloroform-methanol-water at 56:38:10. Bands were visualized by spraying with α-naphthol, ninhydrin, or Dittmer-Lester reagent and then charring.

### SRM assay.

A single-reaction monitoring (SRM) assay was developed for detection and quantification of HMBPP using a Waters Xevo TQ-S triple quadrapole mass spectrometer (MS) with electrospray ionization coupled to a Waters Acquity ultraperformance liquid chromatography instrument (UPLC). Specifically, material was separated using a ZIC-pHILIC stationary phase (Merck Millipore; 150 by 2.1 mm, 5 μM) with a decreasing gradient of acetonitrile. The gradient was as follows: 0 min of 90% B (100% acetonitrile)–10% A (10 mM ammonium bicarbonate; pH adjusted to 9.6 with ammonium hydroxide), 1.5 min of 90% B–10% A, 8.5 min of 62% B–38% A, and 11 min of 40% B–60% A. The column was washed at 150 μl/min with 5% B for 1 min and then equilibrated under starting conditions with 7.3 column volumes under the initial solvent conditions. The flow rate was 270 μl/min, and the column was held at 50°C. Samples were held at 4°C in the autosampler, and the injection volume was 5 μl. mGLP was injected at 0.526 mg/ml for a total of 2.63 μg loaded onto the column for each neat and HMBPP-spiked injection.

Source and desolvation temperatures were 150°C and 350°C, respectively. Desolvation, cone, collision, and nebulizer gas flows were 850 liters/h, 150 liters/h, 0.2 ml/min, and 7 × 10^5^ Pa, respectively. The MS was operated in selected reaction monitoring mode for the analysis of HMBPP in negative-ion mode with detection of the parent *m/z* for HMB-PP, 261, followed by detection of the most abundant fragments of HMBPP, *m/z* 79 and 97. Argon was used as the collision gas and was operated at a flow rate of 0.2 ml/min. For collision-induced reaction of *m/z* 261 to 79, 12 SRM transitions were monitored, ranging from low (CE = 6) to high (CE = 12) collision energies. For collision-induced reaction of *m/z* 261 to 97, six SRM transitions were monitored, ranging from low (CE = 25) to high (CE = 37) collision energies. The responses from all transitions were summed. All transitions had a dwell time of 10 ms. Collision energy, cone voltage, and dwell time were 18 V, 40 V, and 0.4 s, respectively. Capillary voltage was 2.2 kV.

### γ_9_δ_2_ T cell stimulatory activity.

To expand γ_9_δ_2_ T cells, isolated PBMCs (2 × 10^6^) were cultured with novel antigen fractions or controls (medium rested, 50 pM HMBPP, and 20 μg/ml of MtbWL and live BCG at a multiplicity of infection [MOI] of 0.03). On day 7, the PBMCs were harvested, counted, and stained with anti-γδ TCR, anti-αβ TCR, and anti-CD3. Absolute numbers (AN) of γ_9_δ_2_ T cells were computed by multiplying the flow cytometric percentages by the numbers of viable cells present after expansion. Expansion indices (EI) were calculated as the fold expansion of the absolute number of γ_9_δ_2_ T cells after stimulation with treated lysates compared to the absolute number of γ_9_δ_2_ T cells after rest in medium.

### Assay of γδ T cell-mediated inhibition of intracellular mycobacterial growth.

For most experiments, target and effector cell populations were prepared as previously described (36, 41, 42). Briefly, PBMCs were plated to obtain macrophage targets that were infected with BCG (MOI = 3) the day before addition of T cells. Total antigen-expanded PBMCs and freshly purified T cells were added to infected macrophages at an effector-to-target cell ratio (E:T) of 12.5:1. Medium-rested PBMCs (1.5 × 10^5^/well) were added to support optimal intracellular mycobacterial growth as previously described (42). Residual intracellular BCG was measured by incorporation of [5, 6-^3^H]uridine (PerkinElmer Wallac Inc., Boston, MA; catalog number NET-367). Percentages of mycobacterial growth inhibition were determined as follows: percent inhibition = 100 − [100 × (DPM in the presence of γ_9_δ_2_ T cells) (DPM in the absence of γ_9_δ_2_ T cells)], where DPM is disintegrations per minute. In an effort to normalize data and apply a standard quantifiable metric to specific antimycobacterial activity of γ_9_δ_2_ T cells, we defined specific activity (SA) as (expansion index) × (percent inhibition of intracellular mycobacterial growth)/dry weight of sample.

### TCR blocking assay.

A modification of a previously published method (35) was used to measure Ag-specific induction of intracellular cytokine production and analyze the Vγ9Vδ2 TCR dependency of γ_9_δ_2_ T cell activation. γ_9_δ_2_ T cells were expanded *in vitro* by activation with MtbWL, mGLP, or HMBPP from 2 healthy, purified protein derivative-positive (PPD^+^) donors. Dendritic cells (DC) were infected with the Danish strain of BCG (43) for 2 h. Purified γ_9_δ_2_ T cells were cultured with BCG-infected or uninfected DC at an antigen-presenting cell (APC)/T cell ratio of 1:1.2 in the presence of anti-CD28 and anti-CD49d (both at 1 μg/ml; BD PharMingen). Anti-TCR Vγ9 (clone 7A5; Pierce Endogen, Rockford, IL) and control blocking monoclonal antibodies (MAbs) (R&D Systems, Minneapolis, MN) were added to purified γδ T cells for 30 min at room temperature before coculturing with DC. After 3 h of stimulation at 37°C, 0.7 μl/ml of GolgiStop (BD PharMingen) was added and the cultures were incubated for five more hours at 37°C. Cells were surface stained with anti-CD3, anti-CD4, anti-CD8, and anti-γδ TCR, fixed and permeabilized with Cytofix/Cytoperm (BD PharMingen), and then stained for detection of intracellular IFN-γ, TNF-α, and GzmA before analysis on a FACSCalibur flow cytometer. A minimum of 10,000 events were acquired and analyzed using CellQuest. Percentages of γ_9_δ_2_ T cells responding to the different antigens were defined as the percentages of IFN-γ-, GzmA-, and TNF-α-positive γδ T cells after stimulation.

### BTN3A1 blocking assay.

For BTN3A1 blocking experiments, isolated PBMCs (2 × 10^6^) were cultured with novel antigen fractions or controls (medium rested, 50 pM HMBPP, 20 μg/ml of MtbWL, and 1 μg/ml of mGLP-enriched fractions), in the presence or in the absence of 103.2 anti-CD277 MAb (10 μg/ml), kindly provided by Daniel Olive (Inserm, Marseilles), for 7 days. On day 7, phorbol myristate acetate (PMA)-ionomycin-GolgiStop was added and the cultures were incubated for two more hours at 37°C. Cells were surface stained with anti-CD3, anti-CD4, anti-CD8, and anti-γδ TCR, fixed and permeabilized with Cytofix/Cytoperm (BD PharMingen), and then stained for detection of intracellular Ki-67, IFN-γ, TNF-α, and GzmA before analysis on a FACSCalibur flow cytometer. A minimum of 10,000 events were acquired and analyzed using CellQuest. Absolute numbers of Ki-67-, IFN-γ-, GzmA-, and TNF-α-positive γ_9_δ_2_ T cells were computed by multiplying the flow cytometric percentages by the numbers of viable cells present after expansion.

### Statistical analysis.

Statistical analyses of experimental data, employing nonparametric tests as indicated below, were performed using GraphPad Prism (GraphPad Software, San Diego, CA). For unpaired and paired comparisons, Mann-Whitney U tests and Wilcoxon matched-pairs signed-rank tests were applied, respectively.

## RESULTS

### MtbWL induce the expansion of a protective subset of γ_9_δ_2_ T cells.

**FIG 1:**
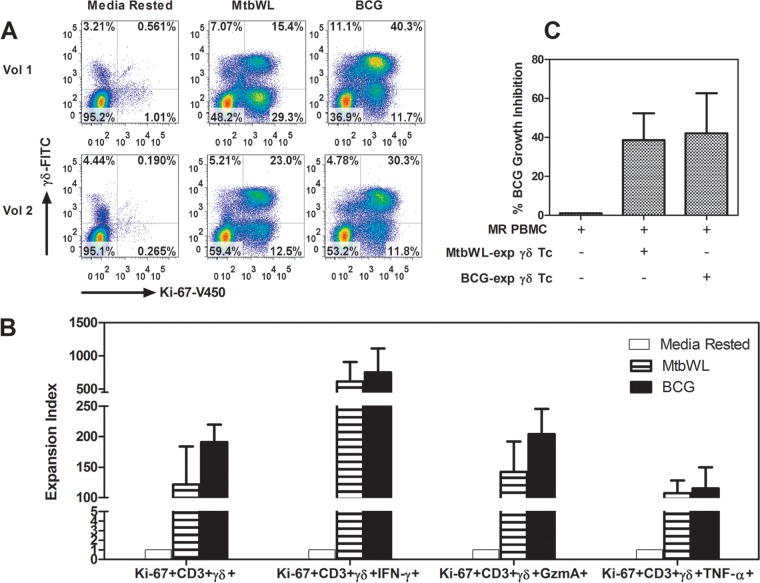
MtbWL can induce the expansion of TB protective γ_9_δ_2_ T cells. (A) MtbWL can expand γ_9_δ_2_ T cells. PBMCs were harvested from 2 PPD^+^ volunteers, 1 week after rest in medium alone or expansion with either BCG or MtbWL. γ_9_δ_2_T cells were identified by staining with anti-CD3, anti-αβ TCR, and anti-γδ TCR. Results shown are gated on CD3^+^ cells. Vol, volunteer. (B) MtbWL and BCG stimulation induced similar levels of effector molecules. PBMCs from two PPD^+^ volunteers were rested in medium (white bars), stimulated with 20 μg/ml of mycobacterial lysate (striped bars), or stimulated with live BCG (MOI, 0.3) (black bars) for 7 days. After 2 h of restimulation with PMA, ionomycin, and GolgiStop, the cells were stained for CD3, CD4, CD8, γδ TCR, Ki-67, intracellular IFN-γ, granzyme A, and TNF-α and analyzed by flow cytometry. (C) MtbWL induce protective γ_9_δ_2_ T cells similar to live BCG infection. PBMC were cultured *in vitro* with BCG or MtbWL or in medium (MR) alone for 7 days and then freshly purified γ_9_δ_2_ T cells cocultured with autologous BCG-infected macrophages at an E:T of 12.5:1. BCG viability was determined 3 days later by [^3^H]uridine incorporation. Both BCG-expanded γ_9_δ_2_ T cells and MtbWL-expanded γ_9_δ_2_ T cells significantly inhibited intracellular mycobacterial growth compared with that in cultures containing medium-rested cells alone.

Our published results demonstrate that human γ_9_δ_2_ T cells circulating among PBMCs obtained from PPD-positive individuals can expand and inhibit intracellular mycobacteria (35, 39, 42). In contrast, we found that polycolonal γ_9_δ_2_ T cells expanded with IPP and other phosphoantigens were unable to inhibit intracellular mycobacteria (35). Therefore, our recent screening strategy to reveal γ_9_δ_2_ T cell antigens focused on the identification of M. tuberculosis fractions that not only expand γ_9_δ_2_ T cells but also expand γ_9_δ_2_ T cells with the ability to inhibit intracellular mycobacteria. We first verified that expansion of protective γ_9_δ_2_ T cells did not require infection of host cells with viable mycobacteria by comparing the ability of MtbWL-expanded γ_9_δ_2_ T cells and live BCG-expanded γ_9_δ_2_ T cells to inhibit intracellular mycobacterial growth. Similar preparations of MtbWL-expanded and live BCG-expanded γ_9_δ_2_ T cells produced IFN-γ, TNF-α, and granzyme A (GzmA) (Fig. 1A and B). Importantly, MtbWL-expanded and live BCG-expanded γ_9_δ_2_ T cells displayed similar functional capacities to inhibit intracellular mycobacterial growth (Fig. 1C). These results clearly demonstrate that MtbWL contains antigens capable of inducing the expansion of a protective subset of γ_9_δ_2_ T cells.

### Mycobacterial components stimulating inhibitory γ_9_δ_2_ T cells are protease resistant but sensitive to acid hydrolysis.

**FIG 2:**
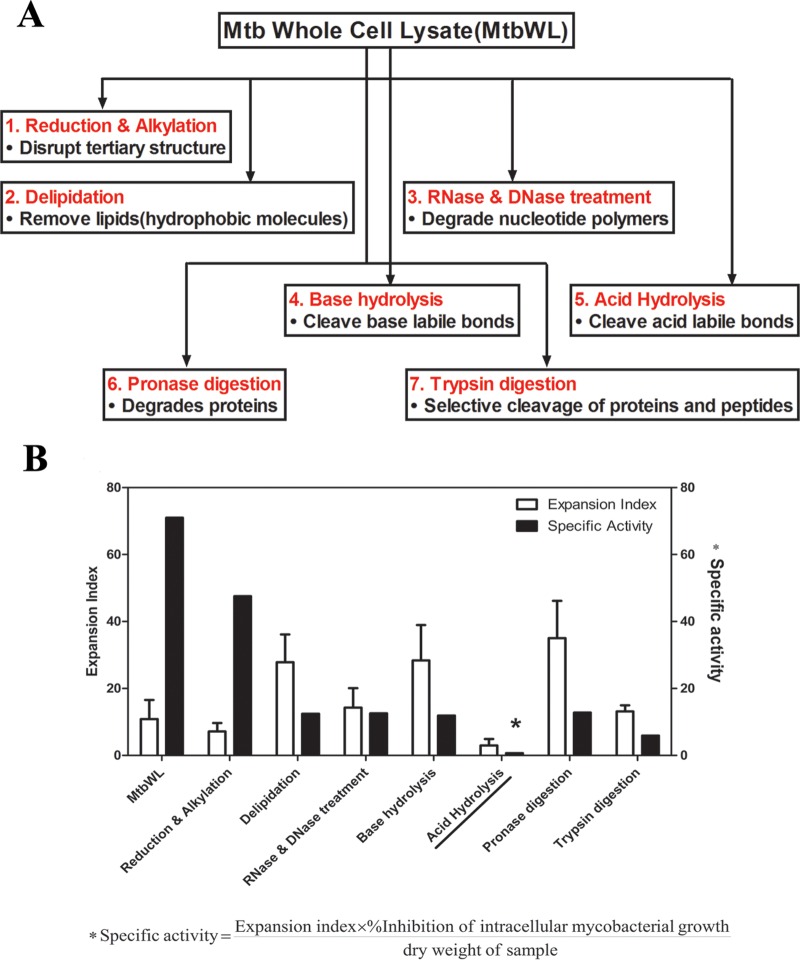
Initial unbiased evaluation of nature of MtbWL. (A) Mycobacterial lysates were treated in order to remove large classes of molecules. The initial biochemical treatments consisted of (1) reduction and alkylation to disrupt disulfide bonds, (2) delipidation via organic extraction with chloroform-methanol (2:1) to remove nonpolar lipids, (3) RNase and DNase treatment to degrade nucleotide polymers, (4) base hydrolysis to cleave base-labile bonds, (5) acid hydrolysis to cleave acid-labile bonds, (6) pronase digestion to extensively degrade proteins, and (7) trypsin digestion to more selectively cleave proteins to complement pronase digestion. All the treated mycobacterial lysates were tested for the ability to expand γ_9_δ_2_ T cells capable of inhibiting intracellular mycobacteria. The expansion index was calculated by dividing the absolute number of γ_9_δ_2_ T cells after stimulation with treated lysates by the absolute number of γ_9_δ_2_ T cells after rest in medium. Percent inhibition was calculated as described previously (35). Specific activity (SA) was defined as (expansion index × percent inhibition of intracellular mycobacterial growth)/dry weight of sample. The expansion index and SA are shown in panel B. Mycobacterial antigens stimulating inhibitory γ_9_δ_2_ T cells were highly sensitive to acid hydrolysis.

Based on the above-described results, we initiated detailed biochemical work to further characterize the antigens from mycobacteria capable of inducing optimally protective TB-specific γ_9_δ_2_ T cells. We treated mycobacterial lysates in order to remove large classes of molecules. The initial biochemical treatments consisted of (i) reduction and alkylation to disrupt disulfide bonds, (ii) “delipidation” via organic extraction with chloroform-methanol (2:1) to remove nonpolar lipids, (iii) RNase and DNase treatment to degrade nucleotide polymers, (iv) base hydrolysis to cleave base-labile bonds, (v) mild acid hydrolysis to cleave acid labile bonds, (vi) pronase digestion to extensively degrade proteins, and (vii) trypsin digestion to more selectively cleave proteins as a complementary approach to pronase digestion (Fig. 2A).

Disruption of disulfide bonds in the stimulatory antigens had no significant inhibitory effects on the induction of protective γ_9_δ_2_ T cells by MtbWL, suggesting that these structures are unimportant for biological activity. Delipidation also did not remove biological activity, indicating that the TB-specific γ_9_δ_2_ T cell inducing antigen does not consist primarily of nonpolar lipids.

Nuclease treatment did not affect biological activity of the lysates, indicating that the biological activity does not require nucleotide polymers greater than 2 to 3 bp in length. However, nuclease treatment would not have eliminated the TUBag3 or TUBag4 nucleotide conjugates discussed previously. Protein digestion either with the promiscuous pronase or the more selective trypsin did not significantly alter the biological activity of the lysates, indicating that the antigen of interest is nonproteinaceous. Most proteinase digestions cleave proteins into peptides that are 10 to 20 amino acids long, which remain large enough to be loaded onto MHC molecules. Thus, two proteinases were used to increase the likelihood that MHC binding motifs would be cleaved by at least one treatment. Since no diminution of activity was detected with these proteinase treatments, we concluded that the antigen was not a protein or was so heavily modified (e.g., by glycosylation) that it was inaccessible for protein digestion.

Although base hydrolysis should hydrolyze all ester, *O*-glycosyl, and phosphodiester bonds, including inactivation of the previously identified phosphoantigens (IPP, HMBPP, TUBAg1 to -4, etc.), this treatment had no effect on the biological activity capable of expanding inhibitory γ_9_δ_2_ T cells. However, mild acid hydrolysis completely eliminated the stimulatory capacity of mycobacterial lysates (Table 1 and Fig. 2B). Although mild acid hydrolysis destroys several types of chemical bonds, it was the only treatment used which would be expected to hydrolyze complex carbohydrates, suggesting a role for them in the biological activity. These data are consistent with earlier studies demonstrating that γ_9_δ_2_ T cell-stimulating antigens present in mycobacterial extracts were protease resistant and bound lectin (23, 25, 44, 45).

**TABLE 1:** Ability of treated mycobacterial lysates to expand inhibitory γ_9_δ_2_ T cells

Treatment of MtbWL	Expansion index^*a*^	% inhibition^*b*^	% inhibition × EI	Sp act^*c*^
None	10.84 ± 5.740	71.39 ± 13.03	773.87	71.01
Reduction and alkylation	7.14 ± 2.56	66.63 ± 12.11	475.74	47.55
Delipidation	27.87 ± 8.30	69.04 ± 5.17	1,924.14	12.45
RNase and DNase digestion	14.27 ± 5.820	82.57 ± 2.760	1,178.27	12.58
Base hydrolysis	28.35 ± 10.58	74.5 ± 6.86	2,112.08	11.89
Acid hydrolysis	2.98 ± 1.96	−10.55 ± 0.28	−31.44	0.68
Pronase digestion	35.04 ± 11.15	64.22 ± 6.400	2,250.27	12.81
Trypsin digestion	13.16 ± 1.840	67.76 ± 10.80	891.72	5.90

a Expansion index is calculated as the fold expansion of the absolute number of γ_9_δ_2_ T cells after stimulation with treated lysates compared to the absolute number of γ_9_δ_2_ T cells after rest in medium. Results are presented as means ± SE.

b Percent inhibition is calculated as described in Materials and Methods. Results are presented as means ± SE.

c Specific activity is calculated as (percent inhibition × EI)/dry weight of the material.

### A 6-*O*-methylglucose (6-*O*-Me-Glc) lipopolysaccharide (mGLP) derivative is responsible for expansion of protective TB-specific γ_9_δ_2_ T cells.

**FIG 3:**
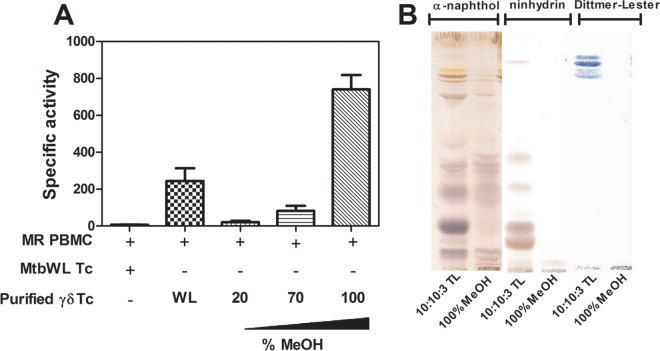
M. tuberculosis polar glycolipids retain the greatest specific activity for induction of γ_9_δ_2_ T cell inhibitory activity. M. tuberculosis H37Rv was cultured, and cells were harvested, irradiated, lyophilized, and enriched in bioactive product by the organic extraction. This extract was loaded onto a silica gel column and products were eluted with an increasing methanol gradient. (A) All the eluted fractions were tested for the ability to expand γ_9_δ_2_ T cells inhibitory for intracellular mycobacterial growth. (B) TLC analysis of biologically active extracts of M. tuberculosis. Shown are TLC results for M. tuberculosis H37Rv total lipid (100 μg) extracted with CHCl_3_-CH_3_OH-H_2_O (10:10:3) and the 100% MeOH fraction (40 μg) eluted from the silica gel column loaded with dry extract. High-performance TLC sheets were developed in CHCl_3_-CH_3_OH-H_2_O (56:38:10), and stained with α-naphthol (carbohydrate), ninhydrin (amino acids), or Dittmer-Lester reagent (phosphate).

**FIG 4:**
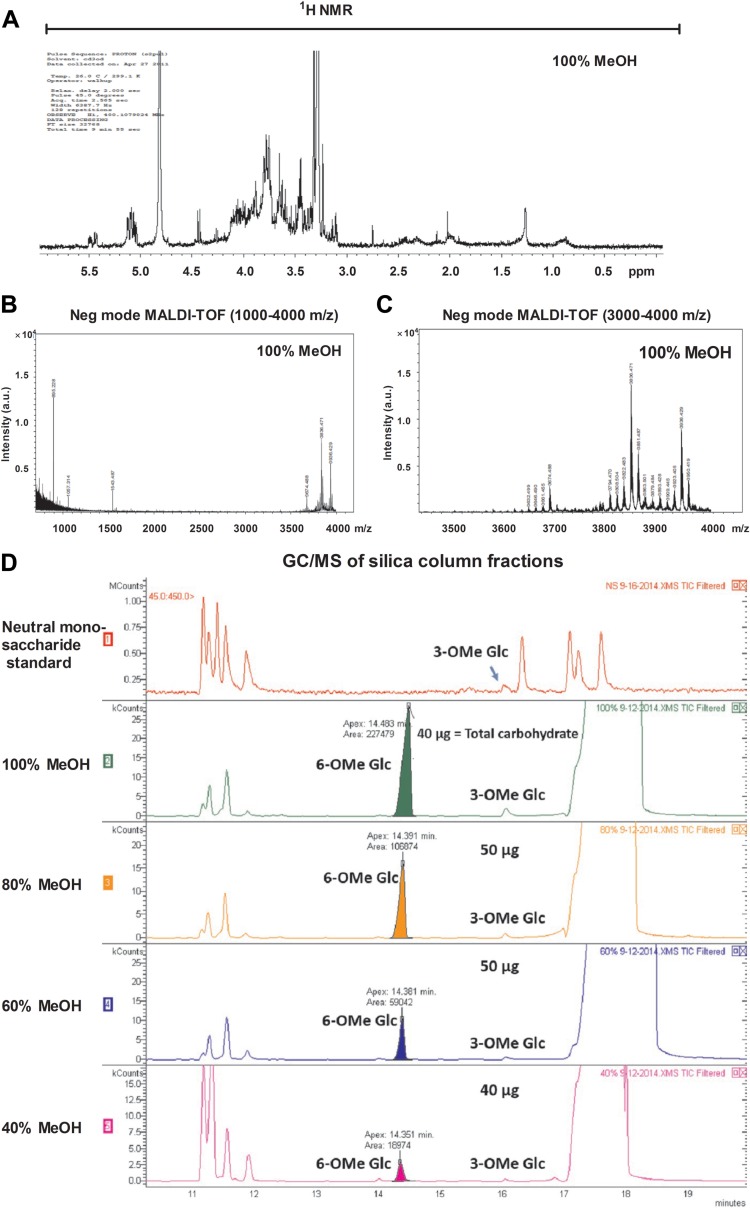
6-*O*-Methylglucose lipopolysaccharide (mGLP), a derivative or similar product thereof, is responsible for expansion of mycobacterium inhibition-specific γ_9_δ_2_ T cells. The 100% MeOH fraction eluted from silica was further analyzed by ^1^H NMR and MALDI-TOF. (A) ^1^H NMR analysis of the 100% MeOH eluate was performed on 4.0 mg of material. NMR spectra revealed a significant presence of *O*-methyl groups and α-anomeric protons corresponding to hexosyl residues. A representative 100% MeOH fraction (1 μl) from a silica gel column loaded with H37Rv total lipid from the chloroform-methanol-water (10:10:3) extraction and eluted with an increasing methanol gradient was mixed with DHB matrix (1 μl) and analyzed in negative electrospray mode. Spectra revealed a high-molecular-mass product in the *m/z* range of 3,600 to 4,000 (B), with peaks separated by 14 amu (C). (D) GC-MS profile of silica gel column fractions from the chloroform-methanol-water (10:10:3) extract: (1) neutral monosaccharide standard, (2) 100% MeOH, (3) 80% MeOH-CHCl_3_, (4) 60% MeOH-CHCl_3_, and (5) 40% MeOH-CHCl_3_.

**FIG 5:**
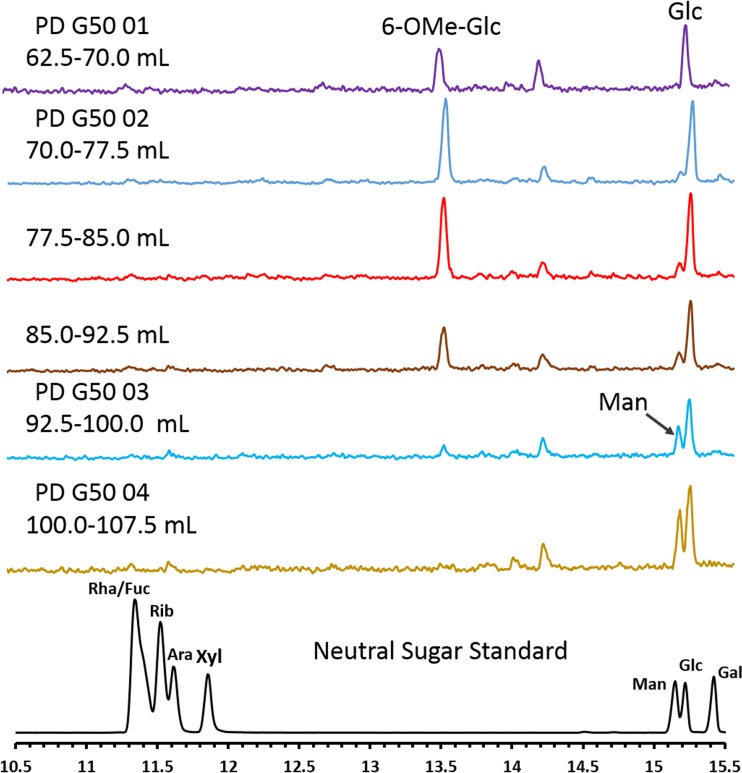
GC-MS of the neutral sugar composition of G-50 column fractions. Shown is the total ion chromatogram of carbohydrate constituents by alditol acetate derivatives on Sephadex G-50 fraction pools; the peak in lines 2 to 7 at retention time 13.5 min is the characteristic peak for 6-*O*-methylglucose, and that at retention time 15.2 min is for glucose. Both are characteristic constituents of mGLP; fraction PD G50 01 without contaminating mannose (Man) was further used for biological studies.

**FIG 6:**
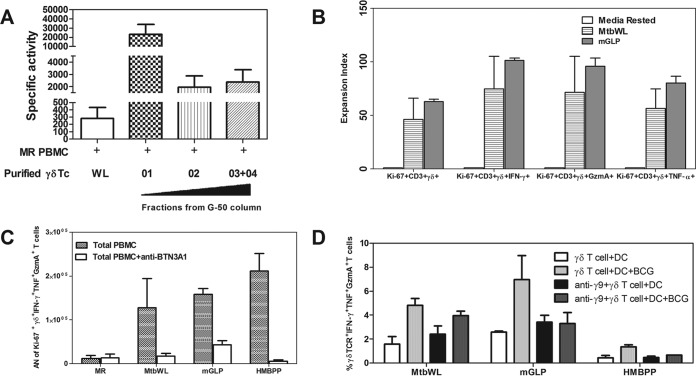
Increased specific activity of purified γ_9_δ_2_ T cells expanded with specific M. tuberculosis G-50 column fractions compared with MtbWL. M. tuberculosis H37Rv was cultured, and cells were harvested, irradiated, lyophilized, and enriched in bioactive product by organic extraction (10:10:3). This extract was loaded onto a silica gel column and products eluted with 100% MeOH (Fig. 3). (A) The 100% MeOH fractions were further loaded onto a G-50 column, and eluted fractions were tested for the ability to expand inhibitory γ_9_δ_2_ T cells. Note the >100-fold increase in SA with a carbohydrate-rich fraction (G-50 column fraction 01) compared with both total MtbWL and the subsequent fraction 02 or a mixture of fractions 03 and 04 eluted off the G-50 column. (B) mGLP and MtbWL induced similar levels of γδ T cell proliferation responses. PBMCs from two PPD^+^ volunteers were rested in medium (white bars), stimulated with 20 μg/ml of MtbWL (striped bars), or stimulated with 1 μg/ml of mGLP-enriched fraction (gray bars) for 7 days. After 2 h of restimulation with PMA, ionomycin, and GolgiStop, the cells were stained for CD3, CD4, CD8, γδ, Ki-67, intracellular IFN-γ, granzyme A, and TNF-α and analyzed by flow cytometry. (C) Blocking BTN3A1, known to be involved in phosphoantigen activation of γ_9_δ_2_ T cells, also inhibits mGLP-induced γ_9_δ_2_ T cell activation. T cells were cultured for 7 days with medium alone, 20 μg/ml of MtbWL, or 1 μg/ml of mGLP-enriched fraction, in the presence or in the absence of 103.2 anti-CD277 MAb (10 μg/ml). After 2 h of restimulation with PMA, ionomycin, and GolgiStop, the cells were stained for CD3, CD4, CD8, γδ, Ki-67, intracellular IFN-γ, granzyme A, and TNF-α and analyzed by flow cytometry. Absolute numbers (AN) of Ki-67-, IFN-γ-, GzmA-, and TNF-α-positive γ_9_δ_2_ T cells were computed by multiplying the flow cytometric percentages times the numbers of viable cells present after expansion. (D) γδ T cells expanded with MtbWL, the mGLP-enriched fraction, or HMBPP were incubated with BCG-infected or uninfected DC for 3 h. A total of 0.7 μl/ml of GolgiStop (BD PharMingen) was added, and cultures were incubated for five more hours at 37°C. IFN-γ, TNF-α and GzmA production was detected by intracellular cytokine staining. γδ T cells were pretreated with anti-TCR Vγ9 or control MAb for 30 min before coculturing with DC. Responses were detected as the percentages of IFN-γ-, GzmA-, and TNF-α-positive γδ T cells after DC stimulation. Results from one representative experiment of three performed are shown.

Our subsequent identification strategy involved testing progressively more purified M. tuberculosis biochemical fractions for the ability to expand γ_9_δ_2_ T cells with inhibitory activity for intracellular mycobacteria. Chloroform-methanol-water (10:10:3) extractions of whole M. tuberculosis cells were found to retain the greatest biological activity. The resulting extract was subsequently loaded onto a silica gel column and products were eluted with an increasing methanol gradient. All eluted fractions were tested for the ability to expand inhibitory γ_9_δ_2_ T cells. The highest biological SA was found in the most polar fractions eluted with 100% methanol (Fig. 3A). A 100% MeOH fraction was analyzed by TLC and stained with α-naphthol to detect carbohydrate, ninhydrin to detect peptide, and Dittmer-Lester to detect phosphate (Fig. 3B). The crude fraction of CHCl_3_-CH_3_OH-H_2_O (10:10:3) starting material (left lanes) and the further-resolved 100% methanol sample (right lanes) demonstrate the complexity of the initial extract and the partial purification success achieved with the downstream 100% MeOH fraction. The consensus analyses identified products rich in hexose residues, devoid of products containing amide bonds or peptides, and with few phosphate and no aromatic (phenyl) residues. Additionally, NMR spectra revealed a significant presence of *O*-methyl groups and anomeric protons corresponding to hexosyl residues (Fig. 4A). MALDI-TOF analysis revealed a series of high-molecular-mass products (∼2,100 to 3,900 amu), with profiles similar to that reported for the spectra of methylglucose lipopolysaccharide (46–48) (Fig. 4B and C). The MALDI-TOF data directly support the data obtained from the NMR spectra and GC-MS analyses of alditol acetate derivatives of the enriched fractions (Fig. 4D). GC-MS analyses corroborated the NMR findings and further identified 3-*O*- and 6-*O*-methylglucose in these highly active fractions. In order to achieve further enrichment of mGLP (estimated by GC-MS [Fig. 5]) from the crude 100% CH_3_OH fraction, the methanol fraction was dried, reconstituted in water, and subjected to size exclusion (Sephadex G-50; MWCO of 1,500 to 30,000) chromatography. γ_9_δ_2_ T cells expanded with the fraction from 62.5 to 70.0 ml, eluting after the void volume of the column (6-*O*-Me-Glc, Glc, and 3-*O*-Me Glc in a ratio of 6:4:1) was found to exhibit the greatest antimycobacterial activity (Fig. 6A). In addition, the results in Fig. 6B demonstrate that the mGLP fractions induce significant proliferative expansion only in γ_9_δ_2_ T cells and not in αβ T cells. Furthermore, Fig. 6C shows that blocking BTN3A1, known to be involved in phosphoantigen activation of γ_9_δ_2_ T cells, also inhibits mGLP-induced γ_9_δ_2_ T cell activation. To determine if the mGLP-enriched fractions induced γ_9_δ_2_ T cells in a TCR-dependent manner, or simply via innate immune activation receptors, we utilized an anti-Vγ9 blocking MAb known to inhibit γ_9_δ_2_ T cell responses induced by IPP and HMBPP. These results confirm that the mGLP fractions contain antigens capable of inducing TCR-dependent responses (Fig. 6D). Based on these findings, we hypothesized that 6-*O*-methylglucose lipopolysaccharide, or a derivative or similar product thereof, is responsible for the biological activity inducing the mycobacterium-inhibitory capacity of γ_9_δ_2_ T cells.

### The novel mycobacterial stimulatory molecules are distinct from phosphoantigens.

**FIG 7:**
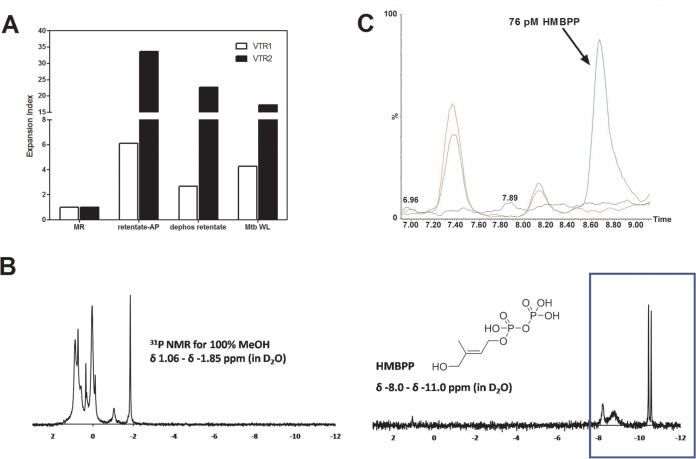
The novel mycobacterial stimulatory molecules are distinct from phosphoantigens of H37Rv. Total lipid (10:10:3) was dried via rotary evaporation and resuspended in chloroform-methanol (2:1). The suspension was transferred to glass tubes and centrifuged. The chloroform-methanol-insoluble material was resuspended in water and filtered through an Amicon Ultra (MWCO, 3,000) iteratively to obtained both retentate and eluate substrates for alkaline phosphatase (AP) treatments and dephosphorylated treatments. All the treated fractions were tested for the ability to expand γ_9_δ_2_ T cells. (A) EI of γδ T cells. Active fractions of M. tuberculosis, when treated with alkaline phosphatase to hydrolyze phosphoryl esters, still retain the ability to expand γδ T cells. (B) Difference in ^31^P NMR chemical shifts of 100% MeOH fraction and commercial HMBPP (blue box). (C) Overlay of injection series for solvent only (black line), mGLP only (red line), and mGLP spiked with 76 pM HMBPP (green line). Note the absence of HMBPP (to a limit of detection of 19 pM), which resolves between the retention time of 8.55 and 8.95 min, in mGLP, even when 2.63 μg of sample (green line) is injected onto the MS instrument. Minor peaks represent other mGLP products, resolving separately from HMBPP, during chromatography of mGLP. The *x* axis represents retention time (minutes); the *y* axis is normalized to percent relative abundance of signal in each sample based on the total peak area for 76 pM HMBPP.

Previously identified small antigens known to stimulate γδ T cells include phosphorylated metabolites generated in the methyl erythritol pathway in mycobacteria. The most representative stimulatory molecules are isopentenyl pyrophosphate (IPP) and (*E*)-4-hydroxy-3-methyl-but-2-enyl pyrophosphate (HMBPP). Both are very small molecules, with molecular weights of 246 and 262, respectively, and are composed of one isoprene unit to which a diphoshate is attached. The phosphate groups of IPP are sensitive to terminal phosphatases such as alkaline phosphatase (23, 26) or apyrase. The novel mycobacterial antigenic activity present in mGLP-enriched fractions could not be abrogated by treatment with alkaline phosphatase (Fig. 7A). These results suggested that the γδ T cell-stimulating bioactivity present in the novel mGLP-enriched mycobacterial fractions is not due to phosphoantigens. ^31^P NMR chemical shifts detected by analysis of the 100% MeOH fraction, as well as commercially available and highly pure HMBPP, are shown in Fig. 7B. The 100% methanol fraction did not show spectral peaks consistent with the HMBPP spectra, further supporting our conclusion that the biologically potent molecules in our novel, mGLP-enriched MtbWL capable of expanding protective γ_9_δ_2_ T cell inducing antigen are not IPP or HMBPP. Finally, an SRM MS assay was developed for sensitive and targeted detection of HMBPP. This assay can detect levels of HMBPP in the picomolar concentration range known to activate γ_9_δ_2_ T cells. Our application of improved chromatography and use of our identical final preparation of mGLP in this assay greatly improved the sensitivity of detection (to 19 pM HMBPP; with reliable quantification of 38 pM HMBPP; see Fig. S1 in the supplemental material). With this improved sensitivity, we can confidently conclude that our mGLP preparations were contaminated with <2 pM concentrations of HMBPP, at least 25-fold less than required for efficient γδ T cell stimulatory activity. HMBPP was not detected in the 100% methanol fraction using this targeted assay, confirming that our fractions were not enriched for this phosphoantigen (Fig. 7C).

## DISCUSSION

In humans, there is a unique γδ T cell subpopulation, termed γ_9_δ_2_ T cells (Vγ2Vδ2 T cells), which express T cell receptors (TCRs) comprising Vγ9 and Vδ2 chains (49, 50). γ_9_δ_2_ T cells exist only in primates (both human and nonhuman) and represent a major circulating γδ T cell subset that typically constitutes 65% to 90% of total peripheral blood γδ T cells (43, 51, 52). γ_9_δ_2_ T cells provide a natural bridge between innate and adaptive immunity, rapidly and potently respond to pathogen infection in mucosal tissues, and are prominently induced by both TB infection and BCG vaccination (2, 11–13, 17–19, 53, 54). Therefore, these cells may serve as potent targets for TB immunotherapy. Clinical trials using various γ_9_δ_2_ T cell activating agents, such as aminobisphosphonates and phosphoantigens, are ongoing for treatment of cancer (55–58). Such trials report low toxicity and improved objective clinical outcomes, including stabilization and partial or full remission of advanced-stage metastatic carcinomas of the prostate (59), lung (60), and kidney (61). Therapeutic activation of γ_9_δ_2_ T cells may also prove to be beneficial in numerous other disease settings, due to their potent adjuvant and effector functions in innate and adaptive immunity (32, 58, 62–65). Data from a recent proof-of-concept study indicate that expansion of γ_9_δ_2_ T cells *in vivo* during pulmonary M. tuberculosis infection can limit M. tuberculosis replication and dissemination and attenuate TB lesions in nonhuman primates (3).

As reported previously (35), we found that γ_9_δ_2_ T cells expanded with IPP were unable to inhibit intracellular mycobacteria. These results suggested that differences in activation induced by live BCG versus IPP were responsible for the differences in inhibitory effector function. Detailed follow-up studies failed to identify any differences in effector function between BCG- and IPP-expanded γ_9_δ_2_ T cells. In contrast, TCR spectratypic and sequence analyses of γ_9_δ_2_ T cell lines expanded by multiple rounds of stimulation with dendritic cells either infected with live BCG or pulsed with IPP demonstrated that the TCR CDR3 sequences of γ_9_δ_2_ T cells expanded by BCG and IPP were markedly different. BCG-expanded γ_9_δ_2_ T cell subsets were significantly less polyclonal than IPP-expanded cells. In addition, the BCG-expanded γ_9_δ_2_ T cells were universally responsive to IPP, but only a small fraction of the total IPP-expanded γ_9_δ_2_ T cells were responsive to BCG. Similar results were seen with T cell lines generated from 5 PPD^+^ individuals and with hundreds of T cell clones expanded from a single individual. Furthermore, the more recently identified HMBPP phosphoantigen, a more potent phosphoantigen for γ_9_δ_2_ T cells expressed in M. tuberculosis- and BCG-infected macrophages, also did not induce γ_9_δ_2_ T cells with pathogen-inhibitory activity. Overall, these results clearly demonstrated that BCG-expanded γ_9_δ_2_ T cells represent a subset of IPP-responsive γ_9_δ_2_ T cells and strongly suggest that BCG-expanded γ_9_δ_2_ T cells express a restricted TCR diversity capable of recognizing unique M. tuberculosis antigens expressed by infected host cells distinct from the previously identified phosphoantigens. Based on this understanding, one of our goals has been to identify the unknown M. tuberculosis antigens capable of stimulating γ_9_δ_2_ T cells with optimal TB protective capacity.

Although we have not yet been able to purify to homogeneity the antigen(s) from mycobacteria which stimulate relevant γ_9_δ_2_ T cells capable of mediating protective effector functions against intracellular mycobacteria, we have made significant progress toward this goal. As an initial step, we proved that MtbWL retain the capacity to stimulate γ_9_δ_2_ T cells with mycobacterium-inhibitory activity. We observed that mild acid hydrolysis of mycobacterial lysates removed the inhibitory γ_9_δ_2_ T cell-stimulatory activity. Enriched polar lipid extracts obtained with chloroform-methanol-water (10:10:3) were further separated on a silica column, and products were eluted with an increasing gradient of methanol. Products eluted with 100% methanol were found to possess the highest specific activity when tested in γδ T cell expansion and M. tuberculosis growth inhibition assays. This novel fraction was then analyzed by TLC using α-naphthol, ninhydrin, and Dittmer-Lester staining and was further analyzed by MALDI-TOF mass spectrometry, GC-MS, and NMR. The consensus analyses identified products rich in *O*-methylglucose and other hexoses, devoid of peptides, with either low or no phosphate or phenyl residues, including undetectable levels of HMBPP. HMBPP conjugation to our product is still possible despite the MS evidence to the contrary, especially if such conjugates were minor products and their detection was suppressed due to poor ionization. MALDI-TOF MS analysis revealed a series of high-molecular-mass products (∼2,100 to 3,900 amu), with profiles similar to that reported for the spectra of methylglucose lipopolysaccharide. From these analyses, we hypothesized that 6-*O*-methylglucose lipopolysaccharide (mGLP), or a derivative or similar product thereof, is responsible for expansion of γ_9_δ_2_ T cells with mycobacterium-inhibitory activity.

Mycobacteria produce two cytoplasmic polymethylated polysaccharides in which many of the sugar units are partially *O*-methylated; 3-*O*-methylmannose polysaccharides (MMPs) and mGLP (66). Recent evidence indicates that at least two clusters of genes participate in mGLP biosynthesis in M. tuberculosis. One cluster, Rv3030-Rv3037c, encodes a glucosyltransferase (Rv3032) responsible for the elongation of mGLPs, as well as a putative acetyltransferase (Rv3034c), two putative methyltransferases (Rv3030 and Rv3037c), and Rv3031, which may be involved in branching (48). In addition, a second cluster (Rv1208-Rv1213) encodes putative sugar-modifying enzymes (66), including Rv1208, which is crucial in the initiation of mGLP biosynthesis (47), and Rv1212c, which partially compensates for Rv3032 in elongating mGLP (67).

Indeed, our preliminary analysis and extraction of polar lipids from a ΔRv3032 mutant versus wild-type (WT) M. tuberculosis demonstrated a reduction in the presence of mature mGLP, along with accumulation of lower-mass products (∼2,000 to 3,000 amu), by MALDI-TOF analysis (48). Analysis of the knockout (KO) and WT mGLP extracts for γ_9_δ_2_ T cell expansion demonstrated retention of activity, suggesting that lower-mass variants of mGLP retain biological activity (data not shown), further supporting the role of Rv3032 and Rv1212c possessing compensatory enzymatic activities and limiting our ability to use these knockouts in our studies until further structural requirements for the biologically active compound are identified.

Studies of the mechanistic requirements for antigen recognition by γ_9_δ_2_ T cells have revealed important differences with those for αβ TCR cells. The major subset of human peripheral blood γδ cells express γ_9_δ_2_ TCR heterodimers stimulated by phosphorylated metabolites (commonly called phosphoantigens). Our TCR blocking studies indicate that signaling through the Vγ9Vδ2 TCR is required for activation of γ_9_δ_2_ T cells by our novel mGLP-enriched M. tuberculosis stimulatory molecules. In addition, recent studies have identified butyrophilin (BTN3A1) as a key molecule involved in either presenting or sensing phosphoantigens, necessary for stimulation of γ_9_δ_2_ T cells. Butyrophilin-3A (BTN3A/CD277) is present in humans in three isoforms (BTN3A1, BTN3A2, and BTN3A3) (68, 69). Recent studies have shown that antibodies specific for BTN3A1 could either mimic phosphoantigen-mediated activation of the TCR (antibody 20.1) or abrogate this stimulatory effect (antibody 103.2) (70). Biophysical analysis of the underlying interactions suggested that the 20.1 antibody induced or stabilized a TCR-activating conformation of BTN3A1 (71). Additionally, phosphoantigens and 20.1 antibodies activated the same intracellular signaling pathways in the responding γδ T cells, suggesting a common recognition process (72). Sandstrom et al. demonstrated that BTN3A1 acts as an intracellular sensor of phosphoantigens binding phosphoantigen to a surface pocket located in the intracellular domain termed B30.2 domain. This result was confirmed by structural analysis of B30.2-phosphoantigen crystallized complexes and by mutational analysis (73). In the present work, we have provided evidence that our novel mGLP-enriched M. tuberculosis lipid components also require BTN3A1 recognition for activation of γ_9_δ_2_ T cells. It will be crucial in future studies to elucidate the detailed mechanisms involved in mGLP-mediated activation of protective γ_9_δ_2_ T cells and how precisely BTN3A1 is involved (in either presenting or sensing mGLP stimulatory molecules).

In summary, we have shown that IPP and HMBPP expand γ_9_δ_2_ T cells incapable of inhibiting intracellular mycobacteria in human studies. In contrast, a novel polar lipid fraction of M. tuberculosis, enriched in 6-*O*-methylglucose-containing glucose polymers, has been identified that contains potent stimulatory activity for M. tuberculosis-inhibitory γ_9_δ_2_ T cells. Thus, mGLP derivatives may represent important candidates for immunotherapeutic treatment of drug-sensitive and -resistant M. tuberculosis infection and/or use in novel prophylactic TB vaccine approaches.

## Supplementary Material

Supplemental material
