# Supplementary material for: A Subset of Protective γ9δ2 T Cells Is Activated by Novel Mycobacterial Glycolipid Components
Source: Infect Immun. 2016 Aug 19;84(9):2449–62. doi: 10.1128/IAI.01322-15 (PMC4995917; doi:10.1128/IAI.01322-15)
Supplement: Supplemental material [file supp_84_9_2449__index.html]

Supplemental material 

# A Subset of Protective γ9δ2 T Cells Is Activated by Novel Mycobacterial Glycolipid Components

## Supplemental material

- Supplemental file 1 -

  Fig. S1. LOQ curve for HMBPP in solvent and in mGLP matrix.

  PDF, 75K
