# Supplementary material for: A Subset of Protective γ9δ2 T Cells Is Activated by Novel Mycobacterial Glycolipid Components
Source: Infect Immun. 2016 Aug 19;84(9):2449–62. doi: 10.1128/IAI.01322-15 (PMC4995917; doi:10.1128/IAI.01322-15)

Supplementary Figures legend

Supplementary Figure S1. LOQ curve for HMBPP in solvent (A) and in mGLP matrix (B). The LLOQ for HMBPP in solvent and matrix is 38 pM; the LOD is 19 pM. X axis represents pM of HMBPP; Y axis represents the total peak area for HMBPP.

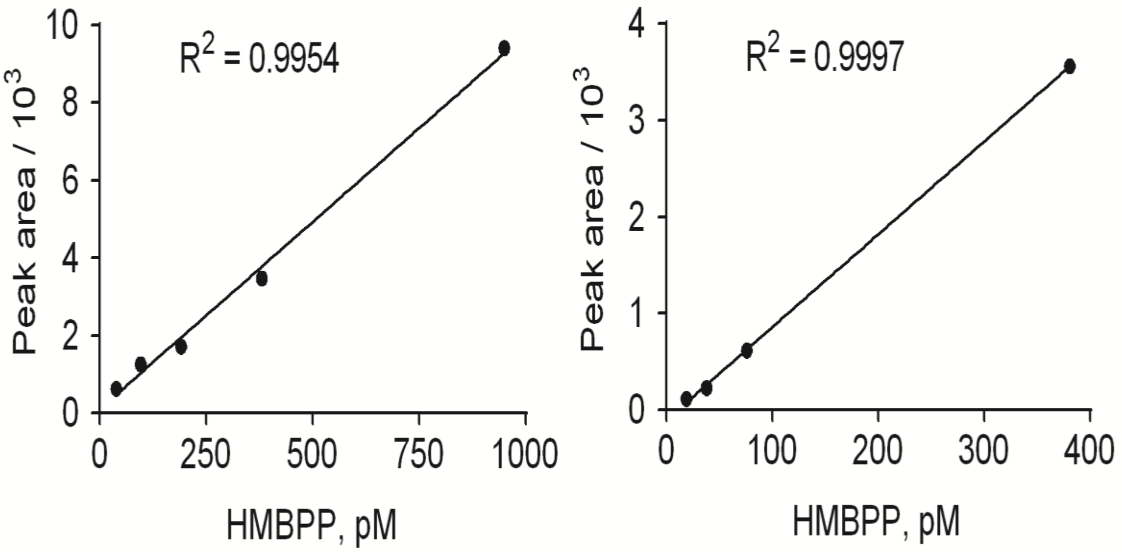

Supplement: Supplemental material [file IAI.01322-15_zii999091787so1.pdf]
